# Mapping the Scientific Landscape Between Respiratory Conditions and Costs: A Bibliometric Analysis

**DOI:** 10.3390/healthcare13182293

**Published:** 2025-09-13

**Authors:** Ioannis Ch. Lampropoulos, Foteini Malli, Eleftherios Aggelopoulos, Angeliki Tsameti, Erasmia Rouka, Zoe Daniil, Konstantinos I. Gourgoulianis

**Affiliations:** 1 Respiratory Medicine Department, Faculty of Medicine, University of Thessaly, 41500 Larissa, Greece; zdaniil@uth.gr (Z.D.); kgourg@med.uth.gr (K.I.G.); 2 Department of Nursing, University of Thessaly, 41500 Larissa, Greece; fmalli@uth.gr (F.M.); errouka@uth.gr (E.R.); 3 Department of Business Administration, University of Patras, 26504 Rio, Greece; eaggelopoulos@upatras.gr; 4 Department of Economics, University of Thessaly, 38446 Volos, Greece; atsameti@uth.gr

**Keywords:** pulmonology, cost, bibliometric review, PubMed, health economics, respiratory diseases, VOSviewer, respiratory medicine

## Abstract

**Introduction:** The objective of the present study was to systematically explore the scientific literature to examine the relationship between respiratory diseases and economic cost. The research question focused on identifying the thematic, methodological, and temporal trends that link these two scientific fields. **Methods:** A comprehensive search was conducted in the PubMed database using the terms “Pulmonology OR respiratory” AND “Cost”, which returned 30,274 publications from 1921 up to April 2025. For the bibliometric review, VOSviewer software was used to create bibliometric maps through the tools of network, overlay, and density visualization. **Results:** The analysis revealed six clusters, which include clinical prognosis, pandemics, pharmacoeconomics, epidemiology, chronic conditions, and health services research. After 2010, there was a particularly important increase in academic research related to pulmonology and cost, with this rise being especially evident during and after the COVID-19 pandemic. Recent studies have increasingly focused on cost-effectiveness, quality of life, hospitalization, and multimorbidity. **Discussion:** The scientific field of respiratory conditions is undergoing a substantial transformation, shifting from traditional clinical descriptions to an interdisciplinary framework that incorporates economic evaluation. This evolution highlights the need for strategies based on economically informed decisions and effective public health policy making. The term “economic cost” in this study refers to both direct costs (e.g., hospitalization and treatment) and indirect economic impacts, such as resource allocation and healthcare burden. **Conclusions:** The findings demonstrate that research linking respiratory diseases and economic cost is expanding rapidly, particularly after the COVID-19 pandemic, and is characterized by interdisciplinary approaches that combine clinical, epidemiological, and economic perspectives. This trend underlines the importance of integrating cost-effectiveness considerations into respiratory healthcare policies and highlights the need for collaborative strategies to ensure sustainable and efficient health systems.

## 1. Introduction

Among the most important public health problems concerns are respiratory diseases, such as Chronic Obstructive Pulmonary Disease (COPD), asthma, and lower respiratory tract infections. Chronic respiratory diseases are globally the third leading cause of death, with approximately 4 million deaths annually, while COPD alone accounted for approximately 212 million cases and 3.3 million deaths in the year 2019 [1]. The authors highlight the need to develop strategies that are cost-effective, particularly in low- and middle-income countries, to ensure the proper management and effective treatment of chronic respiratory diseases. Beyond the public health issues caused by respiratory diseases, they also result in a substantial economic burden. This burden includes not only direct healthcare costs, such as hospitalization and treatment, but also indirect costs, like productivity losses, long-term disability, and caregiver expenses. For example, in the European Union in 2019, the cost of care for patients with chronic respiratory diseases amounted to approximately EUR 380 billion annually [2]. Furthermore, COPD is expected to burden the global economy by approximately USD 4.3 trillion for the period 2020–2050 [3].

Given the growing financial pressures on health systems, strengthening clinicians’ understanding of cost-related issues becomes increasingly important. Organizational changes in healthcare institutions—especially those focused on cost-efficiency—require clinicians to possess both medical and basic financial management skills. Clinicians play a dual role as leaders and managers in driving healthcare reform [4]. However, many medical professionals lack structured education in health economics and systems financing [5]. Addressing this educational gap through training in cost accounting, quality measurement, and resource optimization can improve clinicians’ ability to contribute to sustainable and effective healthcare delivery models.

Based on the above framework, this study’s objective was to investigate the dominant thematic areas, according to the literature, that examine both pulmonary diseases and economic cost. To this end, a search was conducted in the international PubMed database using the terms “Pulmonology OR respiratory” and “Cost”. The search was conducted using the Title/Abstract field, without the use of MeSH terms or any additional filters, such as article type, language, or publication date (beyond the defined period). The use of only the keyword “cost” may have limited the inclusion of studies focusing on economic aspects, such as “cost-effectiveness”, “healthcare expenditure”, or “economic burden”. This limitation is discussed in the corresponding section. Furthermore, no publication-type filters were applied (e.g., editorials, commentaries, or case reports), allowing for a more inclusive review of the literature. The keyword “cost” was selected as a general economic term to ensure broad retrieval of studies. Although terms such as “economic burden” or “cost-effectiveness” are more specific, using “cost” allowed us to maintain thematic inclusivity. This approach is further discussed in the Limitations.

The visualization provided by VOSviewer is expected to illustrate the gradual shift of research interest toward the consideration of cost in pulmonary diseases and the need for interdisciplinary collaborations.

Despite previous bibliometric studies in health economics and respiratory conditions, our study is the first to map cost-related trends exclusively in the field of pulmonology using VOSviewer. This allows for an in-depth analysis of economic dimensions across respiratory subfields, thus addressing a notable gap in the literature.

## 2. Methodology

To conduct a bibliometric review related to the mapping of areas associated with the terms “pulmonology or respiratory” and “cost,” the PubMed database was used. Based on these keywords, the search returned 30,274 publications from the year 1921 up to 2025, specifically up to April 25. No filter restrictions were applied during the search. To analyze the results, the data were exported in nbib format, merged into a single file, and VOSviewer software (version 1.6.20) [6,7] was utilized. The data were useful for selecting analyses based on term contribution, co-authorship, and bibliographic coupling.

Based on the existing data from the PubMed database, we proceeded with a systematic bibliometric mapping of the thematic field using the VOSviewer tool, aiming to visualize the keywords and clusters generated from the total of 30,274 articles.

The PubMed database was selected due to its extensive biomedical coverage and compatibility with VOSviewer software. While other databases, such as Scopus, Web of Science, and EBSCO, also contain literature on health economics, our decision was based on consistency, accessibility, and technical integration with our bibliometric tool. However, we acknowledge that the inclusion of additional databases, such as Scopus or Web of Science, could have enhanced comprehensiveness. Future studies could apply multi-database strategies with defined inclusion and exclusion criteria to ensure broader coverage and comparability.

The VOSviewer tool was selected for this study because it enables the visualization of bibliometric networks based on keywords, co-authorship, and bibliographic coupling. The software is suitable for analyzing large datasets [8], providing excellent visualization of thematic clusters [9]. The program is appropriate for visualizing data and depicting the evolution of research [7].

**Figure 1:**
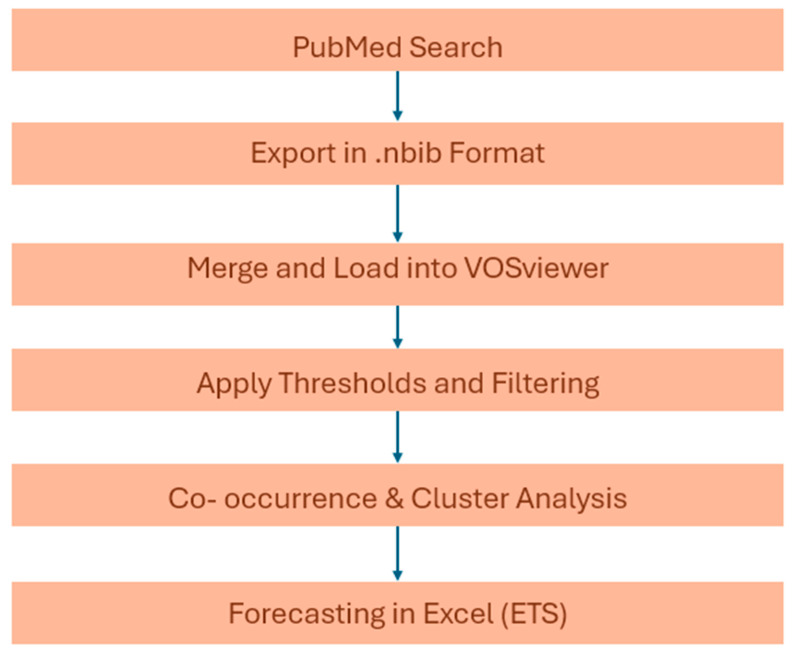
Flowchart of the bibliometric analysis workflow.

Figure 1 provides a schematic overview of the data extraction and analysis process, including the literature retrieval, preprocessing, mapping, and forecasting steps.

Initially, visualization through bibliometric map analysis using the co-occurrence method (co-occurrence of keywords) allowed for the depiction of dominant concepts and their interconnection in thematic clusters. The analysis was conducted using all keywords, and the full counting method was applied [10,11] to account for the total frequency of each word without weighting based on its presence in multiple documents. To ensure that the final output displayed meaningful results, a minimum occurrence threshold of 20 was set, and the 1000 keywords with the highest total link strength were selected. In the VOSviewer interface, we applied a minimum keyword occurrence threshold of 20 to ensure thematic relevance and reduce noise from infrequent or marginal terms. The visualization layout was generated using the default VOSviewer settings for keyword co-occurrence analysis based on the Title/Abstract fields. These settings follow standard practices in large-scale bibliometric studies.

Finally, a filtering process was applied to exclude demographic terms with no thematic relevance to cost (e.g., “middle aged”, “aged”, “male”, “female”). However, terms such as “infant”, “child”, and “preschool” were retained when they appeared in cost-related contexts, especially those concerning vulnerable populations in global health studies. Essentially, all keywords reflecting scientific themes relevant to the research question were retained, such as cost–benefit analysis, hospitalization, risk factors, treatment outcome, and cost of illness, which were deemed pertinent to the subject of this bibliometric review.

After the completion of this analysis, a text co-occurrence analysis was also conducted, functioning as a tool for validation and enrichment of the initial findings in comparison to the previous keyword analysis. This choice enhances the validity of the research and contributes to a deeper understanding of the conceptual layering concerning pulmonary diseases and cost.

Furthermore, to investigate the future development of scientific articles in the coming years, based on the same search terms, Microsoft Excel was used. Forecasting was performed using the FORECAST.ETS function (Microsoft, 2023 [12]), which is based on the Exponential Triple Smoothing (ETS) algorithm. This method estimates yearly predictions using historical publication data, providing upper and lower confidence bounds. The ETS algorithm assumes a linear trend in publication behavior, without adjusting for external factors. We used default seasonal parameters and set the confidence interval at 95%, acknowledging that predictions may be limited by unforeseen disruptions, such as pandemics. Since this version can forecast trends and requires a consistent trajectory, the historical data used covered the years 1970 to 2024, with an estimated forecast reaching up to the year 2041.

The co-occurrence analysis was performed using the full counting method, with a minimum occurrence threshold of 20. VOSviewer software automatically generated clusters based on co-occurrence strength. Thematic interpretation and naming of the clusters were performed manually by the authors. Additionally, non-thematic demographic keywords (e.g., “male”, “female”, “aged”) were manually excluded from the analysis to improve thematic clarity.

To enhance quantitative depth, this review considered standard bibliometric indicators, such as citation counts, contributing countries, and institutional affiliations. However, due to limitations in data export functionalities within the VOSviewer environment, we were unable to include comprehensive tables summarizing the top 10 most cited articles or institutions. Future analyses could incorporate these metrics through the integration of complementary tools or database platforms (e.g., Scopus or Web of Science) that support citation and affiliation-based analytics.

## 3. Results of the Bibliometric Review

### 3.1. Network Visualization

**Figure 2:**
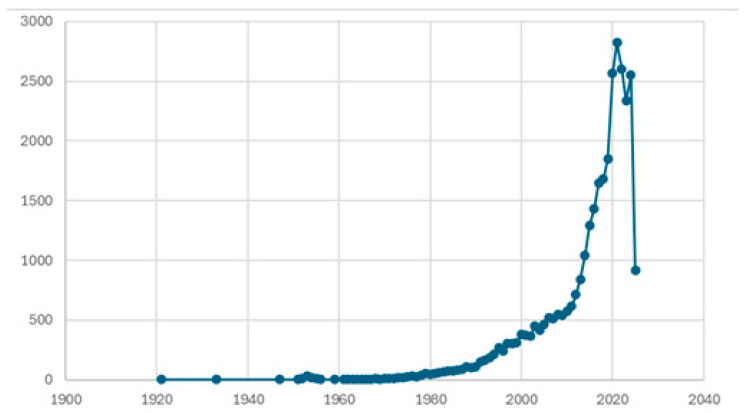
Trend of publications related to the terms “Pulmonology OR respiratory” and “Cost”.

Figure 2 illustrates the increasing trend in publications related to pulmonology and cost, indicating the growing interest in the economic dimension of pulmonary/respiratory diseases.

In the figure, it is observed that from approximately the mid-1990s, there has been a steadily increasing engagement of researchers with cost and respiratory conditions. From 2014 onwards, publications have exceeded 1000 per year, peaking in 2024 with 2552 publications. Regarding the forecasting component, it should be noted that this analysis is intended to be illustrative rather than predictive. Publication trends have been influenced by external factors, such as pandemics, global policy changes, and funding availability, which were not accounted for by the statistical model. Therefore, the projections must be interpreted with caution.

The first bibliometric visualization, which was generated using VOSviewer, mapped the keyword co-occurrence network, capturing the associations among 1000 keywords, which were selected based on the highest total link strength and organized into six thematic clusters.

**Figure 3:**
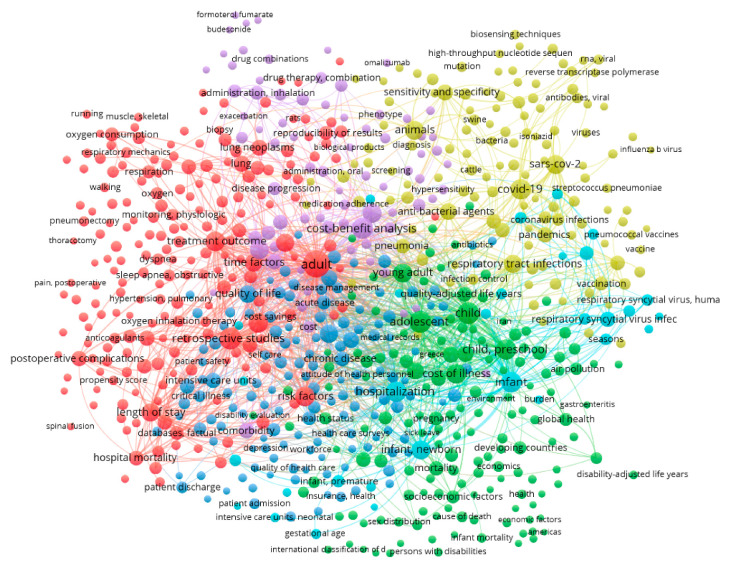
Network visualization. The different colors indicate the six thematic clusters: red = clinical prognosis, green = demographic/socioeconomic, blue = chronic conditions, yellow = pandemics, purple = pharmacoeconomics, and light purple = specialized terms.

In Figure 3, the distribution of keywords among the main thematic clusters can be observed. These clusters are distinguished by color and shape according to their interconnectivity. To improve readability and highlight thematic focus areas, Table 1 below presents representative keywords from each of the six main clusters identified in the co-occurrence analysis. 

The clusters that emerged and are illustrated in Figure 3 are as follows:

The red cluster consists of the largest set of keywords (203 terms) and includes terms such as treatment outcome, length of stay, hospital mortality, retrospective studies, which are related to clinical prognosis, treatment effectiveness, and hospitalization. This red and most dynamic cluster focuses on the cost arising from the clinical and hospital-based aspect of research in relation to cost and pulmonology. For instance, complications and comorbidities in COPD patients substantially increased hospital length of stay, which in turn elevated total hospitalization costs, illustrating the economic burden captured by this cluster [13].

The green cluster includes 149 terms and connects population groups and epidemiological approaches, incorporating terms such as infant, child, preschool, developing countries, mortality. This cluster presents a demographic and socio-economic approach to cost. For example, chronic respiratory diseases place a disproportionate burden on low- and middle-income countries, where children and vulnerable populations face increased morbidity and limited access to care, contributing to both social and economic inequality [14].

The third (blue) cluster includes 138 terms such as chronic disease, hospitalization, quality of life, and cost of illness. This cluster appears to emphasize chronic morbidity and the general economic burden of respiratory diseases. For example, the economic burden of moderate-to-very severe COPD increases substantially with disease severity, exacerbations, and comorbidities, reflecting a direct association between chronic respiratory conditions and escalating healthcare costs [15].

The yellow cluster includes 125 terms and links to topics related to infectious diseases and recent public health crises (COVID-19, pandemics, SARS-CoV-2, influenza), highlighting the pandemic’s role as a major burden factor for both pulmonology and the economy. For instance, it has been estimated that if 80% of the U.S. population was infected with COVID-19, the resulting direct medical costs could reach USD 654 billion, illustrating the extraordinary economic impact that pandemic-related respiratory illnesses impose on healthcare systems [16].

The purple cluster includes 76 more technical and pharmaceutical terms (drug combinations, inhalation administration, biomarkers), reflecting a research focus on areas related to pharmacotherapeutic strategies. For example, a practical framework has been proposed for interpreting cost-effectiveness analyses of pharmacological and biomarker-based interventions in respiratory care, emphasizing their growing importance in guiding resource allocation and policy decisions in constrained healthcare environments [17].

Finally, the smaller light purple cluster includes 30 more specialized terms, which are less connected to the other thematic clusters on the bibliometric map. For instance, utilizing respiratory therapists in clinical pathways has been shown to contribute to both direct and indirect cost reductions, underlining the economic value of specialized respiratory care professionals [18].

**Figure 4:**
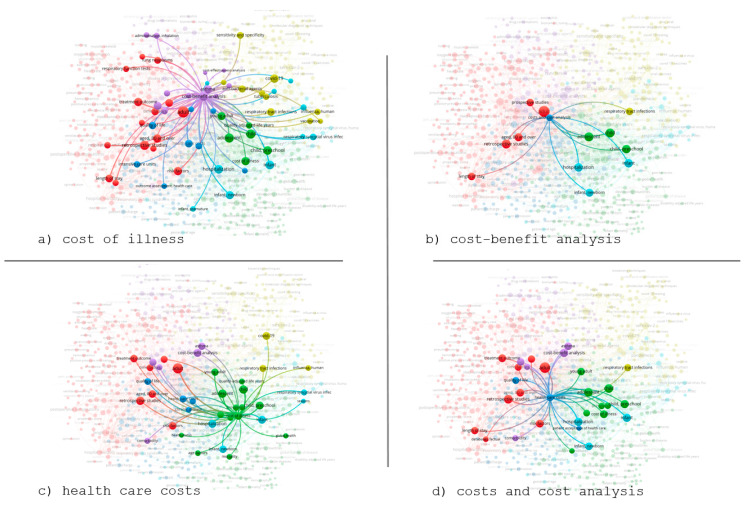
Targeted co-occurrence maps centered on key cost-related terms: (**a**) cost of illness, (**b**) cost–benefit analysis, (**c**) health care costs, (**d**) costs and cost analysis. Colors represent the six thematic clusters: red = clinical prognosis, green = demographic/socioeconomic, blue = chronic conditions, yellow = pandemics, purple = pharmacoeconomics, light purple = specialized terms.

To deepen the understanding of how key economic terms interact across these thematic areas, we created targeted visualizations focusing on four core terms, as illustrated in Figure 4.

Figure 4 shows the spatial positioning of terms, with the connecting lines representing the frequency of co-occurrence among concepts. Thicker lines indicate stronger relationships between terms. Finally, in the network visualization, the central placement of terms such as cost–benefit analysis, hospitalization, and risk factors suggests the important role these topics play in linking all the subfields.

Due to the importance of the network visualization analysis and to clearly illustrate specific associations from the core search terms in PubMed related to cost and pulmonology, Figure 3 presents a focused visualization centered on four selected terms: cost of illness, cost–benefit analysis, health care costs, and costs and cost analysis.

Figure 4 presents targeted visualizations for the four dominant cost-related terms. In Figure 4a, “cost of illness” appears widely interconnected with hospitalization, chronic disease, and quality-of-life measures. Figure 4b highlights how “cost–benefit analysis” relates to retrospective studies, neonatal care, and treatment evaluation. Figure 4c shows “health care costs” aligning with resource allocation in developing countries. Finally, Figure 4d reveals “costs and cost analysis” as a central connector among multiple thematic domains.

The selected keywords appear more frequently in the network and function as bridges between thematic clusters, linking the economic burden to various fields under study, such as pediatric hospitalization, chronic morbidity, effectiveness evaluation, and adult clinical management.

More specifically, the term cost of illness (Figure 4a) shows connections with terms like hospitalization, infant, and mortality, highlighting the socioeconomic and epidemiological character of the studies. The term cost–benefit analysis (Figure 4b) is strongly linked to terms such as asthma, adult, health care costs, reflecting the role of efficiency-related studies in chronic respiratory diseases. In Figure 4c, the term health care costs is connected with terms like comorbidity, quality of life, and hospital mortality, indicating the clinical and administrative dimension of the economic burden. Finally, costs and cost analysis (Figure 4d) is located at the center of the analytical network and acts as a connecting hub between the thematic areas, thus demonstrating the horizontal nature of the concept of cost in the relevant published literature.

These four focused sub-visualizations of the network analysis contribute to a more comprehensive understanding of the concept of “cost” as a cross-cutting axis that permeates the structure of scientific discourse at multiple levels—both clinical and population-based—emphasizing the importance of studying economics in pulmonary diseases.

### 3.2. Overlay Visualization

**Figure 5:**
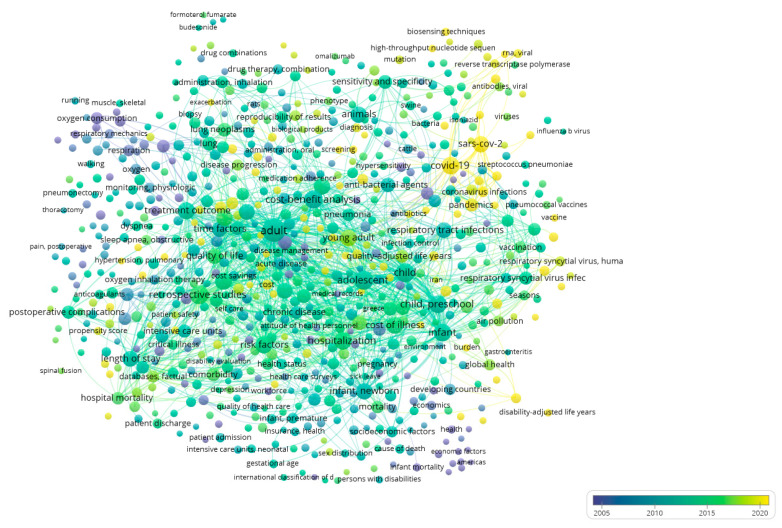
Overlay visualization.

The software also provides overlay visualization (Figure 5), which displays the temporal dimension on the same bibliometric map, showing the average publication year of each term over time.

The blue lines/dots represent older terms, transitioning through green for intermediate ones, and yellow for the most recent terms (newer publications). Through overlay visualization analysis, we obtained a clear indication of the evolution of scientific research on the subject under study. In earlier years, terms such as respiratory distress, retrospective studies, and length of stay (depicted in dark blue) were predominant, indicating the focus of older articles on hospitalization and classical epidemiological approaches. In contrast, concepts such as COVID-19, pandemics, vaccination, and SARS-CoV-2 are clearly more recent, suggesting a shift in research over the past five years toward issues concerning urgent public health crises and their economic impact on society.

Overlay visualization analysis is particularly useful for detecting temporal trends and identifying changes in research priorities. Figure 5 clearly illustrates three chronological transitional phases in scientific research concerning cost in pulmonology and respiratory diseases. In the early research phase, scientists placed emphasis on clinical parameters, studying disease severity in relation to the length of hospital stay. In the second transitional phase, research appears to have shifted to more complex and methodologically supported approaches linked to economic cost evaluation.

Finally, in the third and contemporary phase, and given the SARS-CoV-2 pandemic, research seems to have turned toward associating cost with macroeconomic and epidemiological crisis management.

### 3.3. Density Visualization

**Figure 6:**
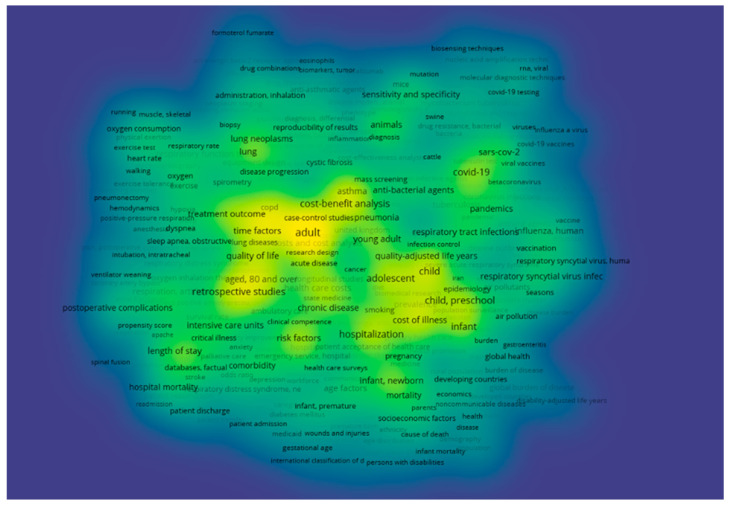
Density visualization. Colors represent the frequency and strength of interconnections: bright yellow = high density (frequently studied terms), green = medium density, and blue = low density (less studied terms).

Finally, Figure 6 presents the density visualization, which visually represents the frequency of term occurrences and the density of their co-occurrences.

The areas depicted in bright yellow represent points with high frequency and strong interconnectivity—essentially the “thematic centers” of the map based on the studied terms. It is worth noting that the regions surrounding the concepts of cost–benefit analysis, hospitalization, chronic disease, and retrospective studies exhibit high density, which demonstrates that these are the central nodes of research activity in the PubMed literature.

In contrast, peripheral terms (depicted in blue or green shades), while still of interest, hold a secondary position in the published studies. Density visualization is particularly useful in highlighting the most saturated areas of the literature, as it provides valuable insights into potential research gaps that require further investigation.

### 3.4. Term Co-Occurrence Analysis

**Figure 7:**
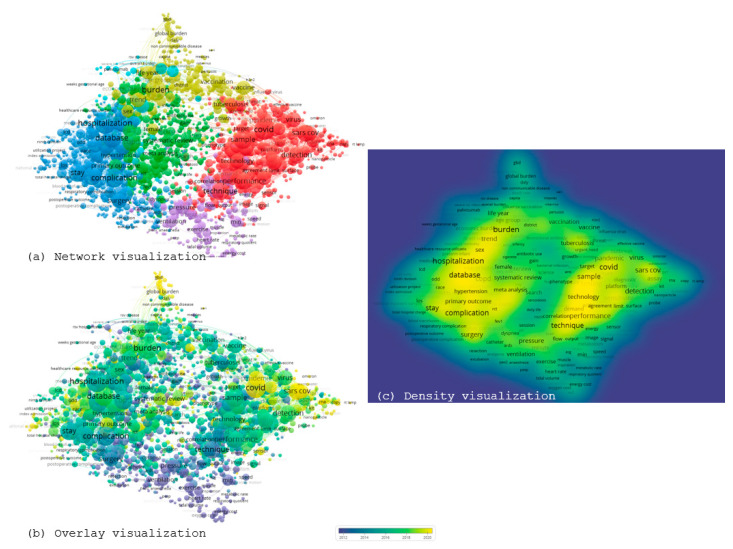
Term co-occurrence in article titles and abstracts (network, overlay, and density visualization). (**a**) Network visualization; (**b**) Overlay visualization; (**c**) Density visualization. Colors indicate thematic clusters: red = clinical prognosis and treatment effectiveness, blue = respiratory conditions and hospitalization, green = demographic/socioeconomic factors, yellow = pandemics and emerging health crises, purple = pharmacoeconomics, and light purple = specialized terms.

In the next stage, a term co-occurrence analysis was performed based on the abstracts and titles of the articles under study. This stage of the work served as a complementary bibliometric approach, enhancing the bibliometric expansion. Term co-occurrence analysis was carried out using the software function “Create a map based on text data,” applying the binary counting methodology, which records the presence of each term without being affected by its repetition within the same text. To ensure clear and comprehensive thematic coverage of the map, a threshold value of 40 was selected, resulting in 2239 relevant terms. This analysis, in comparison with Section 3.3, highlights the thematic axes that emerge from the abstracts and titles of the articles and is not based solely on keywords. Figure 7 presents the results generated by VOSviewer software in network, overlay, and density visualization, based on the processing of abstracts and titles from the 30,274 articles.

The network visualization illustrates the connections between terms organized into thematic clusters, with terms such as hospitalization, database, burden, sample, performance, and COVID serving as central nodes. This specific cluster, based on article titles and abstracts, presents the interrelation surrounding pulmonology and cost. The overlay visualization displays the temporal dimension of the bibliometric map, emphasizing the shift of research focus over time. Terms such as SARS-CoV-2, assay, platform, and pandemic appear in the more recent years (yellow shades), thus reflecting the current interest of researchers, unlike older terms, such as exercise or pressure. Finally, the density visualization presents the frequency and co-occurrence density of the terms. The areas of highest intensity appear around the terms hospitalization, burden, sample, and complication, indicating the new “thematic centers” of contemporary research. Overall, the term co-occurrence analysis based on titles and abstracts demonstrates both the interdisciplinary and multifactorial characteristics that have developed around the study of cost in pulmonology.

**Figure 8:**
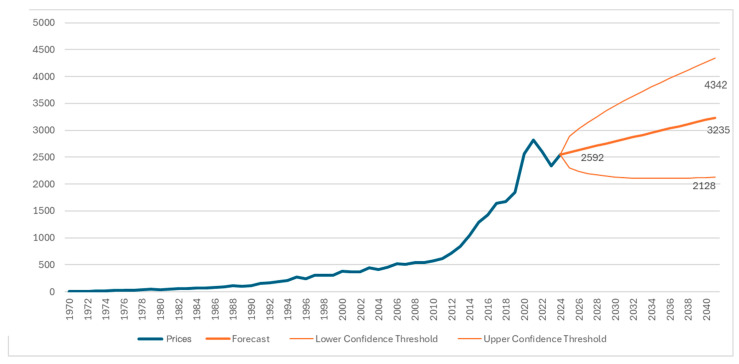
Forecast of publication estimates through 2041.

The results of the forecasting function show an increasing trend in publications related to pulmonology and cost for the years up to 2041 (Figure 8), which was set as the maximum forecast horizon. It is worth noting that this calculation refers to a predictive trend and not a causal estimate. Specifically, as shown in Figure 2, for the year 2025, publications are predicted to be 2592; in 2030, they are expected to reach 2793; and in 2041, the model estimates they will reach 3235, with a lower bound of 2128 and an upper bound of 4342 publications.

This continuously increasing trajectory of scientific publications related to the terms under study confirms the relevance of our research question in addressing the research gap between pulmonary diseases and cost.

## 4. Discussion

From the analysis of the present bibliometric study, the need for interdisciplinary collaboration between health scientists and healthcare professionals emerges. The continuously increasing volume of research work, as documented in the quantitative overview of articles from PubMed (30,274 articles up to 2025), combined with the projected future increase in both subjects until 2041, indicates that the economic dimension of pulmonary diseases has acquired particular significance for the scientific community.

The network visualization, through the six clusters, reveals the breadth of the connection between cost and pulmonology, extending from hospital-related expenses and epidemiology to pharmacoeconomics and pandemic crises. Among the six clusters, the red cluster is of particular interest, linking economic aspects with terms such as treatment outcome and length of stay, confirming previous studies that show prolonged hospitalizations dramatically burden healthcare systems [2]. Alternatively, the yellow cluster, which relates to contemporary research topics, is associated with SARS-CoV-2 and reinforces existing studies on the substantial increase in healthcare expenditures during the pandemic, along with the simultaneous need for designing long-term funding strategies [3].

Moreover, the targeted analysis of cost-related terms (cost of illness, cost–benefit analysis, health care costs, and costs and cost analysis) confirms that the concept of cost functions as a connecting hub, integrating diverse scientific approaches such as chronic morbidity and effectiveness evaluation. This targeted visualization presented in our study is particularly important, as it demonstrates the cross-thematic nature of the cost concept in relation to pulmonology.

The overlay visualization revealed the three distinct chronological phases in the evolution of academic writing from the PubMed database, with the initial period focusing on traditional epidemiological studies, the middle period on economic evaluations, and finally the recent period connected with pandemic phenomena and public health, with the emergence of the SARS-CoV-2 pandemic acting as a defining factor. This transition toward terms such as “vaccination” and “SARS-CoV-2” reflects the immediate adaptation of scientists to socio-economic conditions and, as seen in the visualizations, supports our finding that economic science functions as an intermediary field connecting with the medical sciences.

The density visualization confirmed the high research density around concepts related to cost and pulmonology, as demonstrated by terms such as hospitalization, cost–benefit analysis, and chronic disease. Moreover, the visualization also depicts areas of low density, which could serve as a basis for further research—particularly in connections between cost and prevention or between pulmonary diseases and social inequalities. Finally, the text data co-occurrence analysis, based on the titles and abstracts of the 30,274 articles, highlights new terms such as “database”, “sample”, and “assay”, which could indicate the emergence of a new field related to technology and digitalization in the study of pulmonary diseases and associated accounting/economic expenditures. This analysis demonstrates methodological validity, reinforcing the findings of the earlier keyword analysis and thus enhancing the coherence of our conclusions.

Future studies could expand this analysis by incorporating cost-effectiveness modeling, patient-level economic data, or comparative bibliometric mapping across multiple chronic diseases. Furthermore, longitudinal studies may investigate how economic discourse evolves in response to health crises or policy reforms.

### Theoretical and Practical Implications

From a theoretical perspective, our study contributes to understanding the interdisciplinary interface between clinical respiratory research and health economics. Empirically, it offers insight into priority areas for cost-effective healthcare policy and resource allocation, highlighting the increasing importance of economic considerations in respiratory disease research.

## 5. Limitations

This study has several limitations. First, it relied solely on the PubMed database, which may have excluded relevant studies indexed in other major bibliographic databases, such as Scopus, Embase, or Web of Science. This database restriction could limit the comprehensiveness and introduce selection bias. Second, the search strategy used only the term “cost” and did not include broader or more specific economic-related terms, such as “cost-effectiveness,” “economic burden,” or “healthcare expenditure.” While the choice of a single inclusive term was intended to enhance consistency, it may have reduced sensitivity and excluded thematically relevant studies. Third, no inclusion or exclusion criteria or filters (e.g., by publication type or study design) were applied. As a result, records such as editorials, commentaries, and case reports were included, potentially introducing heterogeneity in methodological rigor and relevance. Fourth, the exclusive use of PubMed may have introduced language bias by underrepresenting non-English literature and may also reflect a biomedical disciplinary skew due to its indexing policy. Fifth, bibliometric tools, such as VOSviewer, rely on author-provided metadata (e.g., keywords, titles, and abstracts), which may lead to omissions or misclassifications in cases where thematic relevance is not explicitly labeled. For example, important economic aspects may be present in full texts but not in indexed terms. Additionally, keyword-based co-occurrence methods have limited ability to detect nuanced or emergent research topics without manual refinement. Finally, the forecasting model employed (Microsoft Excel’s FORECAST.ETS function) is based on historical publication trends and does not account for external disruptive factors (e.g., global pandemics, shifts in funding, or policy changes). Therefore, it should be interpreted as a trend estimation tool rather than a robust predictive model. Another limitation is potential language bias, as PubMed predominantly indexes English-language journals. Furthermore, keyword-based analyses may not fully capture contextual meaning or underlying conceptual themes, especially when author keywords are inconsistently used.

## 6. Conclusions

In conclusion, the academic articles retrieved from PubMed related to the terms “pulmonology or respiratory” and “cost” reveal a multifaceted and dynamically developing scientific field. The present research could leverage the emerging trend to foster interdisciplinary collaboration across economics, health sciences, and information science. Such integration may offer strategic directions that incorporate all parameters necessary to respond to the vast socio-economic transformations currently underway. Despite the rich literature and the large volume of data recorded over the years linking pulmonology with cost, the connection between the two and how it is shaped in the international academic context remains unclear.

Future research may explore prevention strategies through an economic lens, assess the impact of social inequality on respiratory disease costs, and apply similar bibliometric methods to other disease areas for comparative purposes. Moreover, the role of digital health innovations in reshaping the economic burden of pulmonary care warrants further investigation.

Declaration of generative AI technologies in the writing process:

During the preparation of this work, the authors used ChatGPT version 4.0 to check spelling, grammar, and language, aiming to improve readability. After using this tool/service, the authors reviewed and edited the content as needed and take full responsibility for the content of the publication.

## Figures and Tables

**Table 1:** Representative keywords from each thematic cluster in the co-occurrence network.

Cluster Color	Representative Keywords
Red (Clinical Prognosis)	treatment outcome, length of stay, hospital mortality, retrospective studies, intensive care units
Green (Demographic/Socioeconomic)	infant, child, preschool, developing countries, mortality
Blue (Chronic Conditions)	chronic disease, hospitalization, quality of life, cost of illness, comorbidity
Yellow (Pandemics)	COVID-19, pandemics, SARS-CoV-2, vaccination, respiratory tract infections
Purple (Pharmacoeconomics)	drug combinations, inhalation administration, biomarkers, sensitivity and specificity, pharmacotherapy
Light Purple (Specialized Terms)	pulmonary function test, spirometry, ventilation, exercise test, hemodynamics

## Data Availability

The data used in this study were retrieved from the PubMed database using publicly available search terms. All bibliographic data analyzed are available from PubMed (https://pubmed.ncbi.nlm.nih.gov/ (accessed on 25 April 2025)) and were processed using VOSviewer software (version 1.6.20). No additional datasets were generated or analyzed during the current study.
